# Does the combination of resistance training and stretching increase cardiac overload?

**DOI:** 10.6061/clinics/2019/e1066

**Published:** 2019-08-27

**Authors:** Gabriel Costa e Silva, Roberto Simão, Rodrigo Rodrigues da Conceição, Pablo B. Costa, Humberto Miranda, Rodolfo Rodrigues da Conceição, Roberto L Almeida, Mônica Akemi Sato

**Affiliations:** I Programa de Pos Graduacao em Ciencias da Saude, Faculdade de Medicina do ABC, Centro Universitario Saude ABC (Fundacao ABC), Santo Andre, SP, BR; II Grupo de Pesquisa em Ciencia do Movimento Humano, Colegio Pedro II, Rio de Janeiro, RJ, BR; III Laboratorio de Fisiologia e Desempenho Humano (LFDH), Universidade Federal Rural do Rio de Janeiro (UFRRJ), Seropedica, RJ, BR; IV Faculdade de Educacao Fisica e Esportes, Universidade Federal do Rio de Janeiro (UFRJ), Rio de Janeiro, RJ, BR; V Programa de Pos Graduacao em Endocrinologia Clinica, Universidade Federal de Sao Paulo (UNIFESP), Sao Paulo, SP, BR; VI Department of Kinesiology, California State University, Fullerton, United States

**Keywords:** Static Stretching, Resistance Training, Autonomic Nervous System, Heart, Blood Occlusion

## Abstract

**OBJECTIVES:**

To compare the effects of combinations of resistance training (RT) and static stretching (SS) on heart rate (HR), systolic pressure (SBP), diastolic pressure (DBP), rate pressure product (RPP), oxygen saturation (SpO_2_), rating of perceived effort (RPE), and heart rate variability (HRV) in men.

**METHODS:**

Twelve normotensive healthy men participated in four protocols: a) SS+RT, b) RT+SS, c) RT, and d) SS. Variables were measured before, immediately after, and 15, 30, and 45 min after the sessions.

**RESULTS:**

The combination of SS and RT increased (*p*<0.001) HR when compared to the effects of the noncombined protocols (from 2.38 to 11.02%), and this result indicated metabolic compensation. Regarding DBP, there were differences (*p*<0.001) between the RT and SS groups (53.93±8.59 *vs*. 67.00±7.01 mmHg). SS has been shown to be able to reduce (*p*<0.001) SpO_2_ (4.67%) due to the occlusion caused by a reduction in the caliber of the blood vessels during SS compared to during rest. The increase in RPP (6.88% between RT and SS+RT) along with the HR results indicated higher metabolic stress than that reflected by the RPE (combined protocols increased RPE from 21.63 to 43.25%). The HRV analysis confirmed these results, showing increases (*p*<0.01) in the LF index between the combined and noncombined protocols. Compared to the effect of RT, the combination of SS and RT promoted a vagal suppression root mean square of the successive differences (RMSSD) index (from 9.51 to 21.52%) between the RT and SS+RT groups (*p*<0.01) and between the RT and RT+SS groups (*p*<0.001).

**CONCLUSION:**

Static stretching increases cardiac overload and RPE, reducing oxygen supply, especially when performed in combination with RT.

## INTRODUCTION

Exercises and activities involving muscle stretching have historically been performed before and after physical activities, with the objective of achieving gains in exercise performance and reducing injury risk 1,2. Nevertheless, according to the main studies in the reviewed literature 1,2,3,4, there seems to be no scientific support for this assumption because stretching exercises do not seem to reduce the risk of injury or improve performance. Pope et al. 3 noted that there is no scientific evidence for conclusive statements demonstrating acute or chronic prophylactic effects of stretching. Furthermore, Costa et al. 4 suggested that stretching may increase the risk of injury because it can reduce force and generate muscular imbalances. Stretching may provide neural and/or structural modifications, reflecting changes in neuromuscular performance, as well as in the behavior of physiological variables 5,6.

Lima et al. 5 examined the cardiovascular effects of stretching using the static stretching (SS) method, and they reported that SS increased heart overload by raising the heart rate (HR) (by approximately 20 bpm^-1^) and rate pressure product (RPP) (by approximately 5000 mmHg.bpm^-1^). In addition, previous studies 6-8 have found that pre-exercise SS diminishes the performance of force and may lower the oxygen supply to the stretched muscle 9,10. Silveira et al. 11 reported that SS increases blood pressure and pulse pressure, which indirectly reflects an increase in arterial stiffness in healthy young men.

Farinatti et al. 12 examined whether the cardiovascular changes caused by SS were capable of modulating the autonomic nervous system (ANS) and reported that stretching exercises in men with low flexibility increased sympathetic drive during exercise, whereas the vagal modulation was enhanced in the postexercise period. However, there are remaining doubts regarding the responses in participants with adequate levels of flexibility, as well as the effects of SS in combination with other types of exercise. Thus, studies proposing to understand the autonomic regulation of the cardiovascular system as a function of stretching exercises are of great importance for the science of exercise prescription since such exercises are extremely widespread in sports and the clinical environment with various objectives 13.

Resistance training (RT) has been largely recommended as a nonpharmacological intervention from a prophylactic perspective and to treat cardiovascular dysfunctions and disorders 14. In contrast, SS increases sympathetic activity during exercise 12, and RT can also induce a higher modulation of sympathetic drive in the heart 14. Despite the wide diffusion of SS exercises in combination with other activities, such as RT or sports practices, little is known regarding the risks and consequences of this type of prescription, and there are gaps in the scientific literature regarding cardiovascular and autonomic responses resulting from the combination of RT and stretching exercises. In addition, the order of this combination (stretching before or after) is still subject to many uncertainties.

Therefore, the present study aimed to compare the acute effects of different combinations of RT and SS on HR, systolic blood pressure (SBP), diastolic blood pressure (DBP), rate pressure product (RPP), oxygen saturation (SpO_2_), rating of perceived exertion (RPE), and autonomic responses in normotensive men. Therefore, we hypothesized that the combination of SS and RT can evoke a synergistic effect on autonomic modulation, enhancing the increase in sympathetic activity during exercise, which could be a risk for hypertensive subjects or individuals with other cardiovascular dysfunctions.

## MATERIALS AND METHODS

### Participants

Twelve (n=12) untrained normotensive healthy men (age: 22.3±2.5 years; mass: 76.5±2.7 kg; height: 173.6±9.2 cm; BMI: 25.6±3.1; flexibility assessed by the bank of Wells: 26.7±8.8 cm; HR_rest_: 68.4±7.4 bmp^-1^) participated voluntarily in the experiment. The sample size was estimated using G * Power 3.1 software 15. Based on the software analysis, a sample size of 12 participants was calculated. The sample size was calculated based on Beck procedures 16. The exclusion criteria were history of injury or limited strength or flexibility, hyper or hypomobility, smoking, sedentary lifestyle, and the use of nutritional or pharmacological ergogenic aids.

### Ethical details

All participants read and signed a consent form, and this study was approved by the local ethics committee (CAAE: 78635817.6.0000.0082).

### Procedures

**Figure 1:**
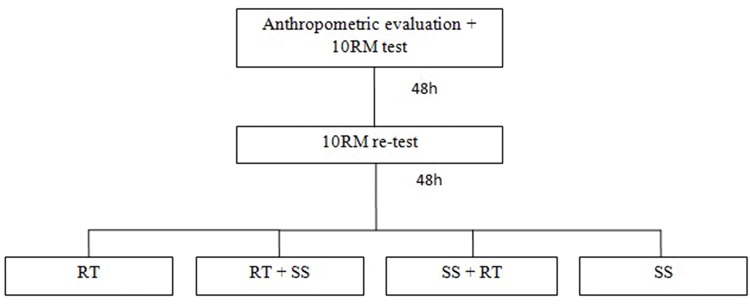
Experimental design. RT = Resistance Training; SS = Static Stretching; RM = Maximal Repetitions.

The present study was performed in a total of 6 visits on nonconsecutive days (48h intervals) that always occurred at the same time, according to Figure 1. On the first visit, the individuals signed the informed consent form and underwent an anthropometric evaluation and a 10RM test for the bench press and leg extension. On the second visit, 10RM retests were performed (both exercises presented excellent reliability between the values of the test and retest, with intraclass correlation coefficient (ICC) values between 0.90 and 0.99).

From the third to the fifth visit, the participants were randomly divided with alternating and counterbalanced entry into the four experimental situations: A) 3 sets of 10 repetitions of the bench press and leg extension (80% 10RM), with 2-min intervals between sets and exercises; B) 3 sets of 10 repetitions of bench press and leg extension (80% 10RM), with 2-min intervals between sets and exercises, preceded by 2 sets of 30 seconds of SS of the pectoral and quadriceps musculature with 40-second intervals between sets (SS + RT); C) 3 sets of 10 repetitions of bench press and leg extension (80% 10RM), with 2-min intervals between sets and exercises, followed by 2 sets of 30 seconds of SS of pectoral and quadriceps musculature with 40-second intervals between sets (RT + SS); and D) 2 sets of 30 seconds of SS of the pectoral and quadriceps musculature with a 40-second interval between sets (SS). All study visits took place in the laboratory, where the relative air humidity (55-60%) and ambient temperature (20-25° C) were controlled. The individuals were instructed not to consume any alcoholic beverages or caffeine before performing the protocols and to maintain their eating habits throughout the period of the research.

The SBP, DBP, RPP, SpO_2_, and RPE variables were measured at rest and immediately after, 15 min after, 30 min after, and 45 min after the end of the session. HR and HRV (root mean square of the successive differences (RMSSD), low frequency (LF), high frequency (HF), and LF / HF) were measured 1 min after the end of the session.

### 10RM test

To minimize the margin of error in the 10RM test, familiarization and standardized instructions were adopted before the test, such that all the evaluated participants were aware of the entire routine involved in the data collection 17. The evaluator was instructed on techniques for performing the bench press and leg extension exercises, being aware of the position adopted by the practitioner during the tests since small variations in the positioning of the joints involved in the movement can trigger other muscles, leading to erroneous interpretations of the obtained scores (an elastic band was used to limit the amplitude of movements). Additionally, 48h after the first day, a retest was performed to examine the maximum load reliability (10RM).

### Stretching protocol

The stretching protocol consisted of two 30-second sets of SS of the pectoral and quadriceps musculature 18, with a 40-second interval between sets. The stretching of the pectorals was performed passively with horizontal shoulder flexion at 90° according to Costa e Silva et al. 9. The same procedure was followed for the stretching of the quadriceps muscles; the participants remained in a pronated position with stabilized hips 6. The knee flexion and hip extension were performed to the maximal point of discomfort. These exercises were performed bilaterally. Participants reported values above 8 on a scale (0-10) proposed by McCully 10 to indicate the maximal stretching supported 10.

### Resistance training protocol

The resistance protocol consisted of the execution of 3 sets of 10 repetitions of the bench press exercise (upper limb exercise) with free weights at 80% 10RM and 3 sets of 10 repetitions of the leg extension exercise (lower limb exercise) also at an intensity of 80% 10RM, with a 2 min interval between sets and exercises. To guarantee technical quality during the exercises, the same standard procedures of the 10RM test were used to perform the movements.

### HRV and HR

The data for HR analysis were obtained using a heart rate monitor (Polar, RS 800 CX, USA) validated in previous important studies 19,20. The electrode was moistened and positioned directly on the sternum (at the level of the xiphoid process). After obtaining the data, they were transferred to a computer by Polar^®^ infrared interface (Polar Precision Performance 4.03 software, Polar Electro OY, Kempele, Finland) and stored in the computer for future analysis. The HR was measured before (at rest), 1 min after, and at 15, 30, and 45 min after the different experimental protocols. The data were then exported to the Kubios HRV (Kubios HRV Analysis Version 3.0 beta, Finland) program, where they were filtered according to the recommendations of the Task Force of Spectral Analysis 21. The spectral analysis in the domain of frequency was performed by the Fourier algorithm transform. The HRV indexes were analyzed according to the parameters of LF in normalized units (LF-nu), which provides information about the activity of the sympathetic nervous system; HF in normalized units (HF-nu), which provides information about the activity of the parasympathetic nervous system; and the standard deviation (SD) of differences between adjacent normal R-R intervals (RMSSD), which provides information regarding the predominance of the parasympathetic or sympathetic nervous system after Fourier transformation and noise filtering. The LF/HF ratio was determined to evaluate the sympathovagal balance.

### Blood pressure and RPP

The measurement of SBP and DBP values was performed using an ambulatory blood pressure monitor (ABPM; Burdick 90217 Ultralite, USA) using the oscillometric technique 22, thus enabling automatic recording of blood pressure values. SBP and DBP were also measured before, immediately after, and at 15, 30, and 45 min after the different experimental protocols. To obtain the RPP values, the HR values were multiplied by the SBP values (RPP=HR×SBP).

### RPE

The participants were instructed to rate their perceived effort using an RPE scale. Borg's perceived effort rating scale is a scale that ranges from 06 to 20 23. The prediction of RPE is a quantitative method of analyzing the individual during physical effort tests or exercise sessions. The scale was applied at the end of each experimental protocol.

### Oxygen saturation

To obtain oxygen saturation values, a finger pulse oximeter (Nonin Onyx 9500, USA) was used. Finger pulse oximetry can be considered an indirect measure of oxygen consumption 24. To obtain the data, the probe was fixed on the index finger of the dominant hand, which was supported on a fixed surface for stabilization. SpO_2_ was measured before, immediately after, and at 15, 30, and 45 min after the different experimental protocols.

### Statistical analysis

A Shapiro-Wilk test was used to verify the normality of the data. The reliability between test and retest sessions was analyzed using a two-factor random effect model intraclass correlation coefficient (ICC) and paired t-tests. A two-way ANOVA was performed to determine differences in HR, SBP, DBP, RPP, RPE, SpO_2_, and HRV after the experimental treatments. The Bonferroni post hoc test was then performed when appropriate. Statistical analyses were conducted using the statistical software package GraphPad Prism 5.0. The significance level was set at *p*<0.05.

## RESULTS

The respective HR, SBP, DBP, RPP, SpO_2_ and RPE differences are presented in Table 1.

**Figure 2:**
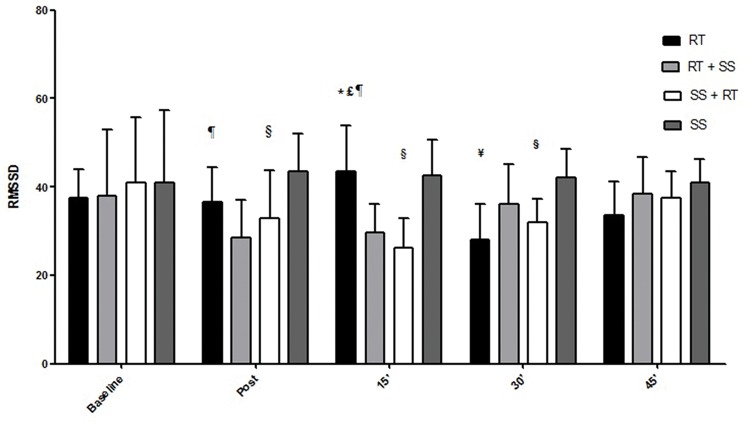
Mean and standard deviation of the values of the RMSSD index (nu) according to the different protocols. * Represents a significant difference (*p*<0.01) between RT and RT + SS 15 min-post. £ Represents a significant difference (*p*<0.001) between RT and SS + RT 15 min-post. ¥ Represents a significant difference (*p*<0.01) between RT and SS 30 min-post. ¶ Represents a significant difference (*p*<0.001) between RT + SS and SS postexercise and a significant difference (*p*<0.01) between RT + SS and SS 15 min-post. § Represents a significant difference (*p*<0.05) between SS + RT and SS postexercise, a significant difference (*p*<0.001) between SS + RT and SS 15 min-post and a significant difference (*p*<0.05) between SS + RT and SS 30 min-post.

**Figure 3:**
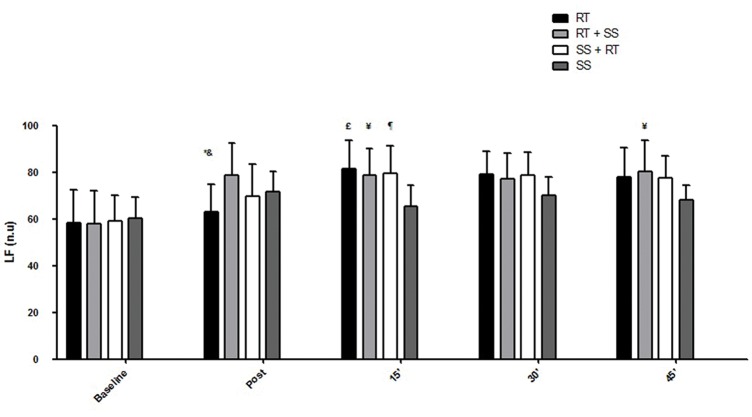
Mean and standard deviation of LF index values (nu) as a function of the different protocols. * Represents a significant difference (*p*<0.01) between RT and RT + SS postexercise. & Represents a significant difference (*p*<0.05) between RT and SS + RT postexercise. £ Represents a significant difference (*p*<0.01) between RT and SS 15 min-post. ¥ Represents a significant difference (*p*<0.05) between RT + SS and SS 15 min-post and a significant difference (*p*<0.05) between RT + SS and SS 45 min-post. ¶ Represents a significant difference (*p*<0.05) between SS + RT and SS 15 min-post.

**Figure 4:**
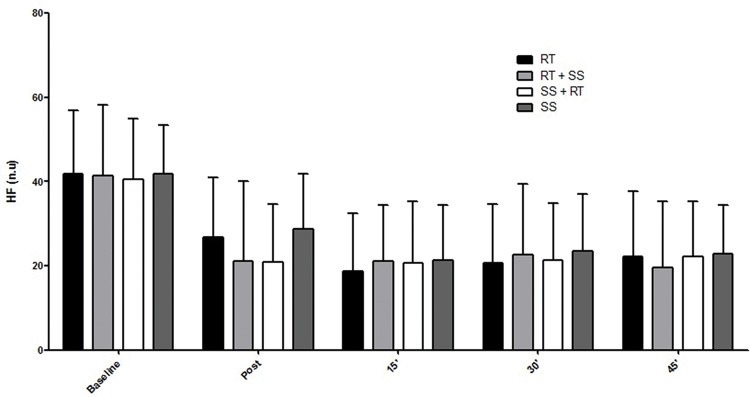
Mean and standard deviation of the HF index (nu) according to the different protocols.

**Figure 5:**
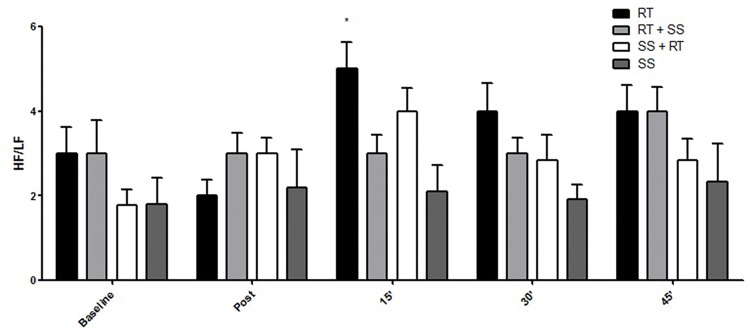
Mean and standard deviation of the LF/HF ratio values as a function of the different protocols. * Represents a significant difference (*p*<0.01) between RT and SS 15 min-post.

Regarding HRV, the respective differences in the RMSSD index, LF index, HF index and LF / HF ratio are presented in the Figures 2-5. 

## DISCUSSION

Our main results indicate that this type of combination (SS and RT) may increase heart overload, raising HR and RPE (Table 1). According to Fowles et al. 25, SS generates less neural activation due to decreased alpha-motoneuron sensitivity and autogenic inhibition. These phenomena caused by the stretching associated with the viscoelastic and musculotendinous characteristics 26 provide less stiffness, causing the subsequent exercise to be more intense, which can cause metabolic and perceptive compensation, as observed in the present study. Accordingly, previous studies 6,8 have shown that SS, when performed in combination with RT, may reduce performance. In addition, SS combined with RT was also shown to be effective in increasing RPP and, according to Table 1, to reduce the oxygen supply due to possible blood occlusion as a function of the properties of the biomaterial tissues of the blood vessels 27.

SS, when performed before RT (SS+RT), generated higher overload than when performed at the end of the session (RT+SS). This result can be attributed to the fact that SS has the lowest intensity, causing the postexercise responses to be lower, according to the HR, RPP, RPE, and autonomic results that show higher mean values of cardiac overload during RT than during SS. RT is well described in the literature for its ability to generate a hypotensive effect favoring blood pressure control 28; however, in the present study, when combined with stretching exercises, RT did not show this effect when compared to the effects of the protocols that did not include stretching. The hypotensive effect occurred after only the protocol involving isolated RT compared to the effects of static stretching, as shown in Table 1. Perhaps the reduction in vessel size after stretching did not allow the appearance of a hypotensive effect. The smaller the vessel size, the higher the intra-arterial pressure. Thus, based on peripheral hemodynamic responses, it seems that prescribing SS combined with RT when the purpose is to preserve the cardiovascular system is not an ideal recommendation.

Compared to the values of SpO_2_, RPE, and autonomic responses, the other variables demonstrated smaller changes between pre- and posttest, although the results were significant, which is especially important for some special populations in which minor cardiovascular variations may be life threatening. These findings could probably have occurred as a result of the intervals between sets and exercises that were set at 2 min for RT and 40 seconds for SS, allowing adequate recovery. Additionally, such peripheral responses were reaffirmed by the analysis of the autonomic nervous system, a central indicator of the nervous system (Figures 2, 3, 4 and 5). HRV analysis demonstrated that the protocols involving stretching were able to cause vagal suppression from the RMSSD index and increase sympathetic modulation through the LF index analysis, although the HF and LF / HF indexes did not present significant differences in general. The sympathovagal balance showed that RT was more intense than SS, as expected.

Gladwell and Coote 29 compared the effects of SS to the effects of isometric RT in relation to HRV and other cardiovascular variables (SBP and DBP) in seven (n=7) young men submitted to 1-min SS (passive) of the plantar flexors and reported that the autonomic responses to the stretching were similar to those observed in response to the isometric RT. According to the authors, stretching raised the HR by inhibiting parasympathetic tone, although it did not significantly alter blood pressure responses. Such effects were possibly due to the activation of mechanoreceptors of small muscle fibers. Therefore, the present findings are somewhat confirmed by data from Gladwell and Coote 29 since the performance of SS in combination with RT during the training session was able to significantly increase HR values for up to 30 min after the session in relation to the resting values.

According to Mizuno et al. 30 and a mechanism called pressor reflex, exercises such as stretching can cause changes in the behavior of the autonomic nervous system, especially by increasing the sympathetic modulation emitted by the nucleus of the solitary tract (NTS) as a result of the muscular afferents, especially of type III fibers, and may cause an increase in cardiac responses. In this context, the high sympathetic and pressure behaviors are mediated, in part, by a reflex of the muscle, which is referred to as the pressor reflex 31. The increase in this function is evoked by small-fiber muscle afferents associated with metaboreceptors, which are activated slowly during ischemic muscle contraction, and by mechanoreceptors, which respond rapidly to muscular deformation from muscle stretching 30. The afferent sensorial information is processed by the nervous system, specifically within the NTS, which regulates the sympathetic modulation drive 32.

When analyzing the literature, the perceived scarcity of studies with the purpose of analyzing the behavior of the independent variables of the present study (different orders of combinations of SS and RT) makes it difficult to discuss the data. McCully 10 analyzed the oxygen saturation of 14 active subjects using infrared spectroscopy. After stretching the gastrocnemius, quadriceps and hamstrings using the static method, the results demonstrated that stretching significantly reduced the levels of muscle oxygen in the quadriceps and hamstrings. According to the author, such findings were due to stretching-induced blood occlusion, a factor that causes ischemia in some muscles. The present study demonstrated that the SS of pectoral and quadriceps muscles, when performed in combination with RT, compromised the muscular oxygen supply in young men and could interfere in the reperfusion of oxygen within 45 min after the session. These findings are in agreement with the results of Costa e Silva et al. 9, who reported an acute reduction in the contribution of peripheral oxygen after stretching using static methods and proprioceptive neuromuscular facilitation in female aquatic sport athletes.

The present study revealed a significant increase in RPP along with a significant reduction in oxygen saturation after the session in which SS was performed prior to RT. Although the RPP values were far from a dangerous level, the large reduction in the oxygen supply from the stretching exercises deserves the attention of professionals in the field. SS probably stretches the blood vessels, reducing the vessel caliber. Neto et al. 33 found that by restricting blood flow during RT, oxygen saturation responses were significantly lower while RPP was significantly higher, which could indicate increased cardiac risk and overload, especially for special groups. Later, Neto et al. 34 reported that restriction of blood flow may also increase oxidative stress. Thus, the effects of the inclusion of SS in combination with RT on oxygen supply were similar to those observed by Neto et al. 33 in a study in which the authors reported restricted blood flow. The physiological mechanisms that explain cardiovascular responses during and after stretching exercises have not been fully elucidated in the literature 12, especially when these exercises are combined with other activities such as RT. Hence, professionals responsible for the prescription and control of physical exercise programs should critically analyze the inclusion of stretching exercises in combination with other activities.

Spranger et al. 35 postulated that restricting blood flow significantly increases the risk of deleterious cardiovascular events. Considering that SS is also able to restrict blood flow 9,10, Silveira et al. 11 recruited 26 normotensive women to participate in a stretching session / class involving a series of 20 seconds of stretching for a period of approximately 45 min and reported that SS can acutely increase pulse pressure. The pulse pressure indirectly reflects arterial stiffness, an important variable in the prognosis of coronary diseases and in the development of deleterious changes in perfusion and adverse cardiovascular events. Thus, although the stretch holding time was much shorter in our experiment, it seems that stretching may generate cardiovascular effects, especially if we also consider our HR, RPP, SpO_2_, and HRV findings. It is important to note that the acute increase in these values would not necessarily indicate an instant increase in arterial stiffness. For example, Kruse et al. 13 suggested that low and moderate intensity stretching does not decrease blood flow at the level of the conduit vessels. However, the authors did not perform a stretching session, only a dorsiflexion exercise.

Araújo et al. 36 examined the effects of a combination of SS and RT in a very similar study design and found controversial results. The authors did not verify the flexibility levels of participants, which may have influenced the results. According to Farinatti et al. 12, despite SS promoting a large sympathetic increase during exercise in subjects with low flexibility, the authors observed good vagal regulation after exercise. However, the increased sympathetic drive during exercise may demonstrate a health risk for hypertensive and cardiopathic subjects. In addition, the study cited 36 stating that the results were due to the fact that trained subjects were analyzed (outside the clinical setting) since it is known that trained subjects have a much faster recovery. In addition to not quantifying the intensity of the stretching, another serious methodological error was to perform stretching until only slight discomfort, unlike that performed in practice by health professionals in general. Important variables such as RPP, SpO_2_ and RPE were also not measured, reducing the power of interpretation and extrapolation of the results.

As previous studies have indicated 5,37, cardiac load is increased by stretching exercises alone. Consequently, when the participants performed SS in combination with RT at 80% (the demand is even higher), the results that were obtained were expected. However, many professionals prescribe stretching exercises combined with RT because they are unaware of their physiological effects, believing it can relax the muscles and reduce the intensity of training. Therefore, the present study is characterized by providing important data with good practical applicability. In this sense, Farinatti et al. 37, after verifying the acute cardiovascular responses to stretching in 22 asymptomatic men, suggested that an increase in cardiac responses during stretching sessions should be avoided in individuals with a high risk of adverse cardiovascular events, given that SS is traditionally performed after RT, when the heart may still be overloaded.

Additional studies involving different stretching types, different intensities, different samples, and muscle mass should be proposed for the extrapolation of the findings. The chronic effects of stretching should also be further examined since Nelson et al. 38 showed that although the acute effects of stretching are detrimental to strength development, the chronic effects (10-week stretching program) may increase the levels of this component of physical fitness and can generate different responses as well as physiological behaviors.

Stretching exercises increase cardiac overload and RPE, causing arterial occlusion that is capable of reducing oxygen supply, especially when performed in combination with RT. The HRV results suggest that the greatest cardiovascular overload generated by SS was marked by increased sympathetic modulation of the ANS. In addition, RT and SS performed separately are also capable of generating changes in the cardiovascular system and the ANS, with the effects of RT being more intense than those of SS. Thus, stretching exercise should be performed with caution or should not be performed in combination with RT, especially in hypertensive subjects or people with cardiovascular disease. Therefore, allied health and strength and conditioning professionals might reconsider prescribing stretching in combination with RT when the goal involves the maintenance of the cardiovascular system. Perhaps these exercises do not need to be removed from exercise programs; however, these forms of exercise should be included only when necessary, especially for participants with high heart disease risk and/or established cardiovascular diseases. New studies involving different methods and populations, such as the measurement of biochemical indicators and the respective chronic effects of these exercises, are recommended for the extrapolation of data 39.

Finally, the values between training sets would be of great value to aid in the interpretation of the present results. Thus, we emphasize the importance of new studies with similar designs, such as those performed by Farinatti et al. 37 and Lima et al. 5, to analyze the data between training sets and exercises.

## AUTHOR CONTRIBUTIONS

Costa e Silva G and Conceicao Rodrigo R performed the data collection, manuscript writing and editing, and data analysis. Conceicao Rodolfo R performed data collection and manuscript editing. Simão R, Costa P and Miranda H were responsible for manuscript writing and editing. Almeida RL and Sato MA conducted the orientation, revised and edited the manuscript.

## Figures and Tables

**Table 1:** Means and standard deviations of HR (bpm-1), SBP (mmHg), DBP (mmHg), RPP (bpm-1. mmHg), SpO_2_ (%) and RPE (scale 6-20) values as a function of the different protocols.

		RT	RT+SS	SS+RT	SS
**HR**	Rest	70.08±2.71	71.00±2.00	70.59±2.61	69.12±3.10
	Postexercise	83.08±2.32^A,B,C^	85.10±2.96	90.00±2.93	80.09±2.55
	15 min-post	82.92±2.87^A,B^	82.60±2.45	79.08±2.17	77.30±2.60
	30 min-post	78.58±2.99^B,C^	81.20±2.44	82.92±2.73	76.73±2.30
	45 min-post	78.17±2.72^C^	77.00±2.45	77.00±2.80	73.40±3.01
	Rest	122.12±18.16	123.51±13.03	115.09±20.34	121.63±13.41
	Postexercise	133.75±12.11	121.07±13.86	125.06±11.46	130.32±11.40
**SBP**	15 min-post	123.75±16.32	113.50±11.20	122.50±17.17	129.50±16.70
	30 min-post	122.62±14.72	119.02±16.72	127.03±17.62	127.84±19.01
	45 min-post	121.37±15.76	113.50±21.75	119.11±18.08	123.66±15.90
	Rest	69.58±10.17	65.52±5.02	68.42±6.77	66.50±5.93
	Postexercise	53.93±8.59^D^	55.66±5.10	57.58±6.82	67.00±7.01
**DBP**	15 min-post	64.08±8.01	60.30±9.67	66.17±11.57	66.43±9.45
	30 min-post	65.17±6.38	62.73±10.74	67.00±11.46	66.67±10.94
	45 min-post	66.67±8.39	63.90±7.17	63.83±8.83	65.91±8.85
	Rest	8205.17±884.77	8755.50±565.96	8619.17±811.19	8407.06±415.71
	Postexercise	11218.92±1137.30^E^	10303.30±794.38	12047.17 ±1145.58	10437.32±829.07^F^
**RPP**	15 min-post	9680.58±1266.74	9365.60±968.52	9781.17±1307.23	10010.35±945.38
	30 min-post	10437.75±898.69	9676.25±988.96	10052.33±1303.62	9809.16±1087.05
	45 min-post	9376.42±1188.19	8757.07±968.09	9340.00 ±1268.85	9076.64±983.7
	Rest	98.08±0.63	97.92±0.30	97.92±0.69	98.01±0.45
	Postexercise	97.00±0.63^G,H,I^	90.33±0.82	96.00±0.54	93.44±1.13
**SpO_2_**	15 min-post	97.58±0.67	98.25±1.00	97.08±0.94	97.76±0.90
	30 min-post	97.67±0.90	98.08±1.15	97.75±0.87	98.00±0.57
	45 min-post	97.45±0.64^G,H^	98.50±1.07	98.33±0.64	98.09±0.81
**RPE**	Postexercise	14.50±2.50^J,K^	14.50±0.75	18.50 ±1.00	10.50±0.25

**A** Represents a significant difference in HR (*p*<0.001) between RT and RT + SS postexercise and a significant difference in HR (*p*<0.01) between RT and RT + SS 15 min-post; **B** Represents a significant difference in HR (*p*<0.05) between RT and SS + RT postexercise, a significant difference in HR (*p*<0.001) between RT and SS + RT 15 min-post and a significant difference in HR (*p*<0.05) between RT and SS + RT 30 min-post; **C** Represents a significant difference in HR (*p*<0.001) between RT and SS postexercise, a significant difference in HR (*p*<0.001) between RT and SS 30 min-post and a significant difference in HR (*p*<0.01) between RT and SS 45 min-post. **D** Represents a significant difference in DBP (*p*<0.001) between RT and SS postexercise. **E** Represents a significant difference in RPP (*p*<0.05) between RT and SS + RT postexercise. **F** Represents a significant difference in RPP (*p*<0.05) between SS and SS + RT postexercise. **G** Represents a significant difference in SpO_2_ (*p*<0.001) between RT and RT + SS postexercise and a significant difference in SpO_2_ (*p*<0.01) between RT and RT + SS 45 min-post. **H** Represents a significant difference in SpO_2_ (*p*<0.05) between RT and SS + RT postexercise and a significant difference in SpO_2_ (*p*<0.05) between RT and SS + RT 45 min-post. **I** Represents a significant difference in SpO_2_ (*p*<0.001) between RT and SS postexercise. **J** Represents a significant difference in RPE (*p*<0.001) between RT and SS + RT postexercise. **K** Represents a significant difference in RPE (*p*<0.001) between RT and SS postexercise.
